# Multiple myeloma presenting as a cervical intraforaminal tumor: A case report and review of literature

**DOI:** 10.3389/fsurg.2023.1011152

**Published:** 2023-01-30

**Authors:** Dragan Jankovic, Darius Kalasauskas, Naureen Keric, Malte Ottenhausen, Florian Ringel

**Affiliations:** Department of Neurosurgery, University Medical Center, Johannes Gutenberg University Mainz, Mainz, Germany

**Keywords:** case report, multiple myeloma, outcome, spine, surgery

## Abstract

Multiple myeloma (MM) is a hematological malignancy with characteristic clonal plasma cell proliferation and production of monoclonal immunoglobulins. Although it can often metastasize to the bony spine, completely extravertebral and extra-/intradural manifestations are exceedingly rare. In this case report, we describe a 51-year-old male patient with cervical extradural and intraforaminal MM who was surgically treated in our department. Clinical findings and radiological images were retrieved from medical records and an imaging system. This unusual localization of MM and similar cases in the literature are reviewed in detail. The patient underwent tumor resection via a ventral approach, and postoperative MRI demonstrated a sufficient decompression of neural structures. No new neurological deficits were observed at subsequent follow-ups. Although 7 cases of extramedullary extradural manifestations of multiple myeloma have been described in the literature so far, this is the first case of intraforaminal extramedullary multiple myeloma located in the cervical spine and treated by surgery.

## Introduction

Multiple myeloma (MM) represents 10% of all hematologic cancers with an annual incidence of 6.6 cases per 100,000 (1). It is characterized by the proliferation of neoplastic plasma cells, producing excessive monoclonal immunoglobulin (Ig) or free light chains (2). Multiple myeloma occurs as an extramedullary disease caused by hematogenous or continuous growth via the bone cortex. The most common site of manifestation of MM is the lower thoracic spine, followed by the lumbar spine. Vertebral destruction is primarily responsible for neurological symptoms, while the most frequent symptoms are pain and radiculopathy (3).

Here, we present a case of a patient with previously diagnosed and treated MM who was diagnosed with an intraspinal extradural tumor with an extension to the neuroforamen, resembling a spinal schwannoma, which finally turned out to be a manifestation of MM. We review the literature for the clinical course and management of this important differential diagnosis.

## Case description

A 51-year-old male patient presented to our department with pain in his left shoulder for approximately 9 months. The patient developed burning and piercing pain and dysesthesia in his left arm for 5 months, which corresponded to the C6 dermatome. His pain medication consisted of hydromorphone and dexamethasone. On admission, the patient was awake and cooperative. Cranial nerve status was normal. Except for the tingling paraesthesia of Dig 1, there were no sensory or motor deficits in the extremities. There were no pathological findings in the standing and gait tests.

The patient was diagnosed with multiple myeloma 18 months ago, with manifestation in the 11th thoracic vertebral body, and treated with vertebral body replacement and dorsal spondylodesis T10–12. The surgery was followed by radiotherapy of the T10–12 up to a total dose of 46 Gy and two cycles of adjuvant chemotherapy (bortezomib–cyclophosphamide–dexamethasone). In the subsequent course, autologous stem cell transplantation was performed without complications.

**Figure 1:**
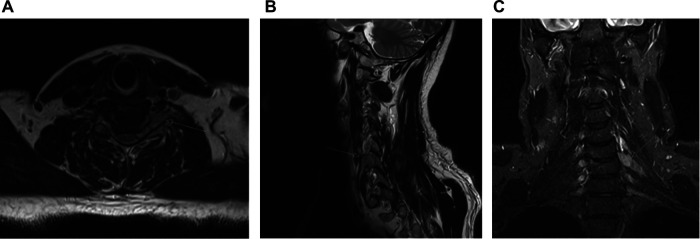
Axial (**A**), sagittal (**B**), and coronal (**C**) post-contrast T1 MRI showing extensive intraspinal, extradural tumor, including extension into the left C5/6 neural exit foramen.

Associated with his new radicular symptoms, an MRI of his cervical spine demonstrated an intraspinal, extradural, and intraforaminal contrast-enhancing lesion adjacent to the left-sided C6 root, without evidence of bone destruction. MRI features were suggestive of a C6 schwannoma (Figure 1).

A blood test showed normal values of hemoglobin (14.4 g/dl, reference range: 13.5–17.5 g/dl) and red cell count (4.72/pl; reference range: 4.2–5.6/pl). Serum LDH value was 275 U/I (reference range: <245 U/I). Tumor resection and decompression of the nerve root were scheduled via a ventral approach. A direct route to the lesion without retraction of the spinal cord was significantly more advantageous with a ventral approach.

As the intraoperative frozen section unexpectedly demonstrated a small blue cell tumor and the tumor diffusely infiltrated the nerve root, the decision was made to perform a partial resection and debulking of the tumor mass. A complete tumor resection could be taken into consideration after the completion of the histological analysis. The patient recovered well from the operation and reported significant pain relief. There were no new neurological deficits. Histologic analysis demonstrated cells with a narrow, poorly demarcated cytoplasm and relatively small, round, and hyperchromatic nuclei with numerous mitoses. The Ki67 proliferation index was >50%. Tumor cells were negative for CD45, CD3, CD20, CD138, synaptophysin, and S10. Positive immunoreactivity was observed only for vimentin antibodies. The final histological analysis revealed a manifestation of MM.

**Figure 2:**
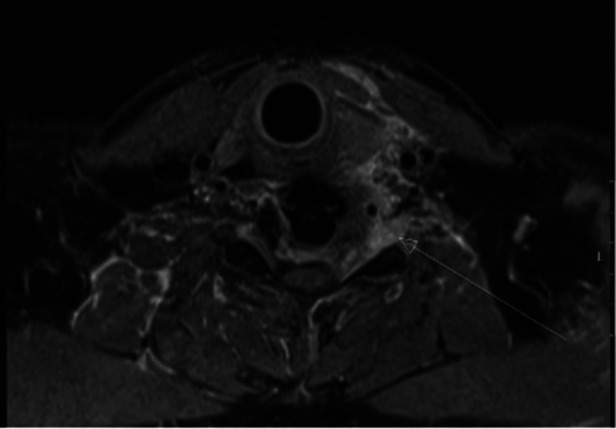
Postoperative axial T1-gadolinium MRI showing partial removal of tumor mass.

Postoperative MRI demonstrated a sufficient decompression of neural structures with a tumor remnant lateral to the nerve root (Figure 2). After an interdisciplinary case discussion, local ablative radiotherapy with a total dose of 45.0 Gy and adjuvant chemotherapy were performed. The patient has been followed for 9 months with no new neurological deficits and no tumor recurrence.

## Discussion

Although MM is the most common malignant tumor in the vertebrae, spinal cord compression occurs in only 5% of cases, usually due to extramedullary epidural tumor growth or a vertebral fracture (4) We searched the PubMed/MEDLINE databases using the following keywords: “multiple myeloma” AND extraosseous AND spinal cord compression. A literature search revealed only 7 cases of extradural manifestation of multiple myeloma (Table 1) (3, 5–10). To our knowledge, this is the first case of intraforaminal extramedullary multiple myeloma located in the cervical spine and treated by surgery. It is postulated that extramedullary hematopoietic (EMH) multiple myeloma arises from hematogenous spread or contiguous seeding from local lytic bone lesions (11). In this case, the manifestation of MM was localized extradural in the lateral spinal canal and neural foramen, without the affection of the adjacent vertebra. The tumor compressed the nerve root, which led to pain, motor, and sensory deficits.

**Table 1:** Published cases of extradural multiple myeloma.

Author	Age/gender	Symptoms	Localization	MRI Signs of Myelopathy	Destruction of vertebral bodies	Intravertebral foramen involvement	Treatment	Follow-up
Avandhani et al. (5)	60/female	Pain, spastic paraparesis	Posteriorly, T6-T7	Yes	No	No	Total resection followed by local radiotherapy and chemotherapy	6 months, improvement of motor power
Hu et al. (3)	45/female	Pain, L5–S1 radiculopathy	L5–S1	NS	No	NS	Surgical resection followed by chemotherapy	Died 5 months later due to pulmonary infection
Okacha et al. (6)	47/male	Progressive paraplegia	Posteriorly, T4–T6	Yes	No	Yes	Subtotal resection	Unchanged
Watanabe et al. (7)	85/male	Paraplegie	C7–T2	Yes	No	No	Chemotherapy	Unchanged
Matsui et al. (8)	52/male	Pain with paresthesie	L3	NS	Yes	Yes	Surgical resection, followed by chemotherapy	Died 5 months later due to respiratory dysfunctionmonths after the onset of symptoms
Lolin et al. (9)	55/female	Pain	T4–5	Yes	No	No		Died 7 months later due to septicemic shock
Palmbach et al. (10)	40/male	Paraplegia	C7–L2/L3	NS	No	Yes	No	Died 3 months after admission due to respiratory insufficiency

It is known that multiple myeloma can be associated with chronic inflammatory demyelinating polyradiculoneuropathy (CIDP) (12). However, several studies have shown that idiopatic CIDP cannot be distinguished from CIDP related to monoclonal gammopathies (13, 14).

Given that pararoteinemia-neuropathy cannot be distinguished from CIDP, their work-up is still a debate. In addition to protein electrophoresis, some authors suggested that nerve biopsy could explain the etiology of neuropathy (15). Peripheral nerve ultrasound has been proposed to differentiate neuropathies associated with antimyelin, CIDP, and M-protein (16). However, due to variable neurotoxic patterns, ultrasound cannot provide relevant results in multiple myeloma.

The primary diagnostic tool for intraspinal tumors is MRI. MM manifests as a contrast-enhancing lesion. These lesions can be diffuse, spreading across multiple spinal levels (17).

The differential diagnoses for intraforaminal and spinal cord lesions usually include schwannoma, neurofibroma, hemangioblastoma, malignant peripheral nerve sheath tumor, spinal metastasis, solitary amyloidoma, and epidural abscess (5). However, the differentiation of intraforaminal lesions based on morphology and intensity may present a challenge for neuroradiologists. As a result, based on MRI features such as T1 iso- to hypointensity and T2 hyperintensity, as well as the configuration, a working diagnosis of schwannoma was made.

The median survival with MM is 2.5 years, while younger patients have a better prognosis. Some studies have reported that the median survival of a patient with EMH MM is 1–11 months (18–20) If the spine is affected, 75% of patients die within 1 year of diagnosis (21). Given the small number of papers published so far, MM's overall survival with extradural localization is unknown. In the cases of epidural extramedullary multiple myeloma reported so far, the cause of death was an infection or respiratory failure.

There are no guidelines for the management of treatment for intraspinal extradural multiple myeloma. In a longitudinal study of 1,003 patients by Varettoni et al., patients with an extramedullary manifestation of multiple myeloma had significantly lower hemoglobin and increased LDH levels (22). Similar findings were reported by studies of Barlogie et al. and Dimopoulus et al., confirming the association between LDH values and a more aggressive course and shorter survival of the patient with MM (23, 24). In our case, the serum LDH value on the day of admission was 275 U/I, which, with a Ki67 finding of >50%, confirms the aggressiveness of the tumor. Due to the extremely high proliferation activity, we hypothesized that MM has an aggressive phenotype, i.e., that there has been a plasmablastic transformation of MM.

An increased incidence of extramedullary relapse has been reported in patients undergoing allogeneic stem cell transplantation. In their retrospective study, Vincent et al. found that the number of previous therapies and age were associated with a higher risk of extramedullary relapse (25). Chemotherapy of extramedullary localizations of MM has so far not yielded satisfactory results (26).

Although extremely rare, a manifestation of multiple myeloma should be included in the differential diagnosis of extradural and intraforaminal tumors in patients with a known MM. Early detection of the lesion is essential, especially if neurological symptoms appear. CSF cytology might be helpful in the diagnosis of intradural lesions, while tumor decompression can prevent neurological deterioration and improve the patient's quality of life. Given that it is difficult to distinguish multiple myeloma from other intraspinal pathology based on signal intensity and morphology on MRI scans, a rapid intraoperative tissue analysis should be considered to avoid unnecessary extensive tumor resections.

## Data Availability

The original contributions presented in the study are included in the article/Supplementary Material, further inquiries can be directed to the corresponding author.
